# Distribution of the amelogenin protein in developing, injured and carious human teeth

**DOI:** 10.3389/fphys.2014.00477

**Published:** 2014-12-10

**Authors:** Thimios A. Mitsiadis, Anna Filatova, Gianpaolo Papaccio, Michel Goldberg, Imad About, Petros Papagerakis

**Affiliations:** ^1^Orofacial Development and Regeneration Unit, Faculty of Medicine, Institute of Oral Biology, ZZM, University of ZurichZurich, Switzerland; ^2^Dipartimento di Medicina Sperimentale, Sezione di Biotecnologie, Istologia Medica e Biologia Molecolare, Seconda Università Degli Studi di NapoliNapoli, Italy; ^3^INSERM UMR-S 1124, Biomédicale des Saints Pères, University Paris DescartesParis, France; ^4^CNRS, Institut des Sciences du Mouvement UMR 7287, Aix-Marseille UniversitéMarseille, France; ^5^Department of Orthodontics and Pediatric Dentistry, School of Dentistry, University of MichiganAnn Arbor, USA; ^6^Center for Organogenesis, School of Medicine, University of MichiganAnn Arbor, USA; ^7^Center for Computational Medicine and Bioinformatics, School of Medicine, University of MichiganAnn Arbor, USA

**Keywords:** amelogenin, ameloblasts, tooth, odontoblast, enamel, carious, dental injury, dental pulp

## Abstract

Amelogenin is the major enamel matrix protein with key roles in amelogenesis. Although for many decades amelogenin was considered to be exclusively expressed by ameloblasts, more recent studies have shown that amelogenin is also expressed in other dental and no-dental cells. However, amelogenin expression in human tissues remains unclear. Here, we show that amelogenin protein is not only expressed during human embryonic development but also in pathological conditions such as carious lesions and injuries after dental cavity preparation. In developing embryonic teeth, amelogenin stage-specific expression is found in all dental epithelia cell populations but with different intensities. In the different layers of enamel matrix, waves of positive vs. negative immunostaining for amelogenin are detected suggesting that the secretion of amelogenin protein is orchestrated by a biological clock. Amelogenin is also expressed transiently in differentiating odontoblasts during predentin formation, but was absent in mature functional odontoblasts. In intact adult teeth, amelogenin was not present in dental pulp, odontoblasts, and dentin. However, in injured and carious adult human teeth amelogenin is strongly re-expressed in newly differentiated odontoblasts and is distributed in the dentinal tubuli under the lesion site. In an *in vitro* culture system, amelogenin is expressed preferentially in human dental pulp cells that start differentiating into odontoblast-like cells and form mineralization nodules. These data suggest that amelogenin plays important roles not only during cytodifferentiation, but also during tooth repair processes in humans.

## Introduction

Sequential and reciprocal interactions between oral epithelium and cranial neural crest-derived mesenchyme result in tooth-specific hard tissues formation (Mitsiadis and Graf, 2009; Jussila and Thesleff, 2012). Epithelial cells differentiate into ameloblasts that synthesize the enamel matrix, while ectomesenchymal cells differentiate into odontoblasts that are responsible for dentin matrix production. Odontoblast differentiation proceeds the differentiation of ameloblasts. Differentiating odontoblasts secrete a collagen-based matrix that forms the mantle dentin, while mature odontoblasts are responsible for the circumpulpar dentin formation (Goldberg et al., 2011). Ameloblast differentiation starts once a short layer of predentin is formed and is followed by enamel matrix deposition and mineralization (Simmer et al., 2010). Enamel formation occurs in the enclosed extracellular space between the ameloblasts and the dentin. This process necessitates a well-orchestrated series of cellular, chemical, and physiological events (Simmer et al., 2010; Mitsiadis and Luder, 2011), and is characterized by three morphologically distinct developmental stages: the secretory, transition, and maturation stages (Smith and Nanci, 1995). During the secretory stage, ameloblasts synthesize and secrete the bulk of enamel matrix that is indispensable to obtain optimal enamel thickness, to initiate mineralization, and to support crystal growth (Simmer et al., 2010).

The main enamel matrix protein is amelogenin, which is secreted and assembled with other less abundant enamel matrix proteins such as enamelin and ameloblastin to form an extensive extracellular framework (Fincham and Simmer, 1997; Robinson et al., 1998). Hydroxyapatite crystallites start to be formed into this framework by the deposition of calcium and phosphate ions (Simmer and Fincham, 1995; Duan, 2014). Once the full thickness of enamel is completed, the mature ameloblasts promote crystal thickening and enamel prism formation. The degradation of amelogenin and other enamel proteins by proteinases such as MMP20 (secretory stage) and KLK4 (maturation stage) is necessary to create the desirable space for crystals growth (Lu et al., 2008). Progressively and under multiple molecular controls enamel maturation is completed and the enamel cementum junction is established (Papagerakis et al., 1999; Bei, 2009; Simmer et al., 2010; Zheng et al., 2014). *Amelogenin* (*AMLX*) mutations in humans have been implicated in amelogenesis imperfecta (AI), a pathology characterized by abnormal enamel formation and organization (Hu et al., 2007; Mitsiadis and Luder, 2011).

Amelogenin has been initially considered to be exclusively expressed by ameloblasts. However, studies during the last decade have shown that amelogenin is also expressed transiently by odontoblasts (Papagerakis et al., 2003), root epithelial cells (Fong and Hammarström, 2000; Janones et al., 2005), and even cells of non-dental origin (Gruenbaum-Cohen et al., 2009). In odontoblasts, *amelogenin mRNA* expression has been detected by *in situ* hybridization during predentin deposition (Papagerakis et al., 2003). Based on the above studies and additional reports it has been suggested that amelogenin may act as a signaling molecule during the initiation of hard matrix formation as well as during the process of tissue regeneration (Veis et al., 2000; Papagerakis et al., 2003). Nevertheless, the role of amelogenin expression in odontoblasts and dentin formation during development and regeneration remains still unclear.

Once teeth are erupted, enamel and dentin composition and integrity might be compromised by carious or traumatic lesions. Parts of the tooth crown could be destroyed by caries or removed during dental treatment such as cavity preparation. Carious decay results to loss of both enamel and dentin proteins and minerals due to the presence of bacteria and to their acidic products (Takahashi and Nyvad, 2008). When caries affect dentin, which is more susceptible in bacteria attacks than enamel (Takahashi and Nyvad, 2008), signaling molecules induce the existing odontoblasts to form a protective layer of dentin called tertiary (or reactionary) dentin.

In dental injuries caused by deep cavity preparations, the damaged odontoblasts are replaced by odontoblast progenitors, which differentiate into new odontoblasts and produce reparative dentin (About et al., 2000a; Heymann et al., 2002). Several studies are undertaken in rodents to understand the process of reparative dentin formation (Sloan et al., 2000; Smith et al., 2001) but only limited studies exist in humans. We have previously reported the role of Nestin, E- and N-cadherin, and Notch2 in reparative dentin formation in human teeth (About et al., 2000b; Heymann et al., 2002; Mitsiadis et al., 2003). However, the potential expression of amelogenin in odontoblasts of developing human teeth, as well as in odontoblasts involved in reparative and reactionary dentin formation has not been studied. The purpose of the present work was to characterize in detail the expression patterns of the amelogenin protein in developing, injured and carious human teeth and to provide a base line for future studies.

## Materials and methods

### Materials

#### Antibodies

A rabbit polyclonal antibody against mouse amelogenin was kindly provided by Dr Pamela DenBesten (University of California, San Francisco, CA, USA). This antibody was demonstrated to react specifically with amelogenin in human tissues in immunohistochemistry (He et al., 2010).

#### Chemicals

Vector Vectastain ABC kit was purchased from Biosys (Compiègne, France). For the preparation of culture media, all materials were purchased from Gibco BRL (Life Technologies Inc., NY, USA). Other chemicals were obtained from Sigma (St. Louis, MO, USA).

#### Culture medium

Minimum Essential Medium (MEM) was supplemented with 10% fetal bovine serum, 2 mM glutamine, 100 UI/mL penicillin, 100 μg/mL streptomycin (Biowhittaker, Gagny, France), and 0.25 μg/mL amphotericin B (Fungizone®).

### Embryonic tissues

Human fetal tissues (18–30 gestational week) were obtained from legal abortions. Fetuses were healthy and all tissues were macroscopically and microscopically normal. Fetuses were fixed immediately by the obstetrician in 10% buffered formalin for 48 h to 5 days according to their size. The samples were decalcified for 3 weeks in formic acid/10% formalin. 10 μm thick sections were used for immunohistochemistry. The maxillary and mandibular processes were embedded in Paraplast at 56°C. This study was carried out in compliance with French legislation, after approval of the Regional Ethics Committee of the Hospital Center of Marseille (CCPPRB Marseille I).

### Cavitated permanent teeth

Cavities were prepared in 10 intact first premolars scheduled for extraction, at the Dental Care Center of Marseille. Cavities 2–3 mm wide and 1–1.3 mm deep were cut into the tooth dentin with a bur. Pulp chambers were not exposed during the preparation of the cavities. The walls of the cavities were immediately conditioned with a 3% hydrogen peroxide solution and dried with an extremely light stream. The cavities were restored with a calcium hydroxide product (Dycal; Dentsplay, USA) that was covered by a temporary filling material (IRM; De Trey Dentsplay IG, Zurich, Switzerland). After a post-operative interval of 4–9 weeks, the teeth were extracted using a local anesthetic after the patient's informed consent.

### Carious permanent teeth

Twenty extracted carious molars of 40 year-old patients were collected for this study after patient's informed consent. The extracted teeth were fixed in 10% neutral-buffered formalin for 24 h, demineralized in sodium formiate for 21 days, and then embedded in paraffin wax. Teeth were serially sectioned (5 μm thick sections) and then processed for immunohistochemistry.

### Cultures of human dental pulp cells

Immediately after extraction, selected healthy premolars scheduled for extraction for orthodontics reasons were washed with sterile phosphate-buffered saline (PBS). The dental pulps were gently removed with forceps, minced with scalpels and then rinsed with PBS. Cultures of human dental pulp cells were performed as previously described (About et al., 2000a). Cells were cultured in the presence or absence of β-glycerophosphate (2 mM) for 4 weeks. After culture, cells were fixed in 4% paraformaldehyde for 1 h at 4°C, and processed for immunohistochemistry.

### Immunohistochemistry

Immunoperoxidase staining of sections and cultured cells was performed as previously described (About et al., 2000a). Briefly, the sections were deparaffinized, exposed to a 0.3% solution of hydrogen peroxide in methanol, and then incubated overnight at 4°C with the primary antibody against amelogenin. The antibody was incubated at a dilution of 1:1000 in PBS containing 0.2% bovine serum albumin (BSA) and 5% normal goat serum (NGS). Peroxidase was detected by incubation with diamino benzidine tetrahydrochloride (DAB) that gives a brown color. After staining, the slides were mounted and observed under a light microscope. In control sections the primary antibodies were omitted. Cultured cells were permeabilized for 15 min with 0.5% Triton X-100 in PBS prior to immunohistochemistry. Sections and cell cultures were then photographed and analyzed for amelogenin expression.

## Results

### Amelogenin expression in the developing deciduous human tooth germs

**Figure 1:**
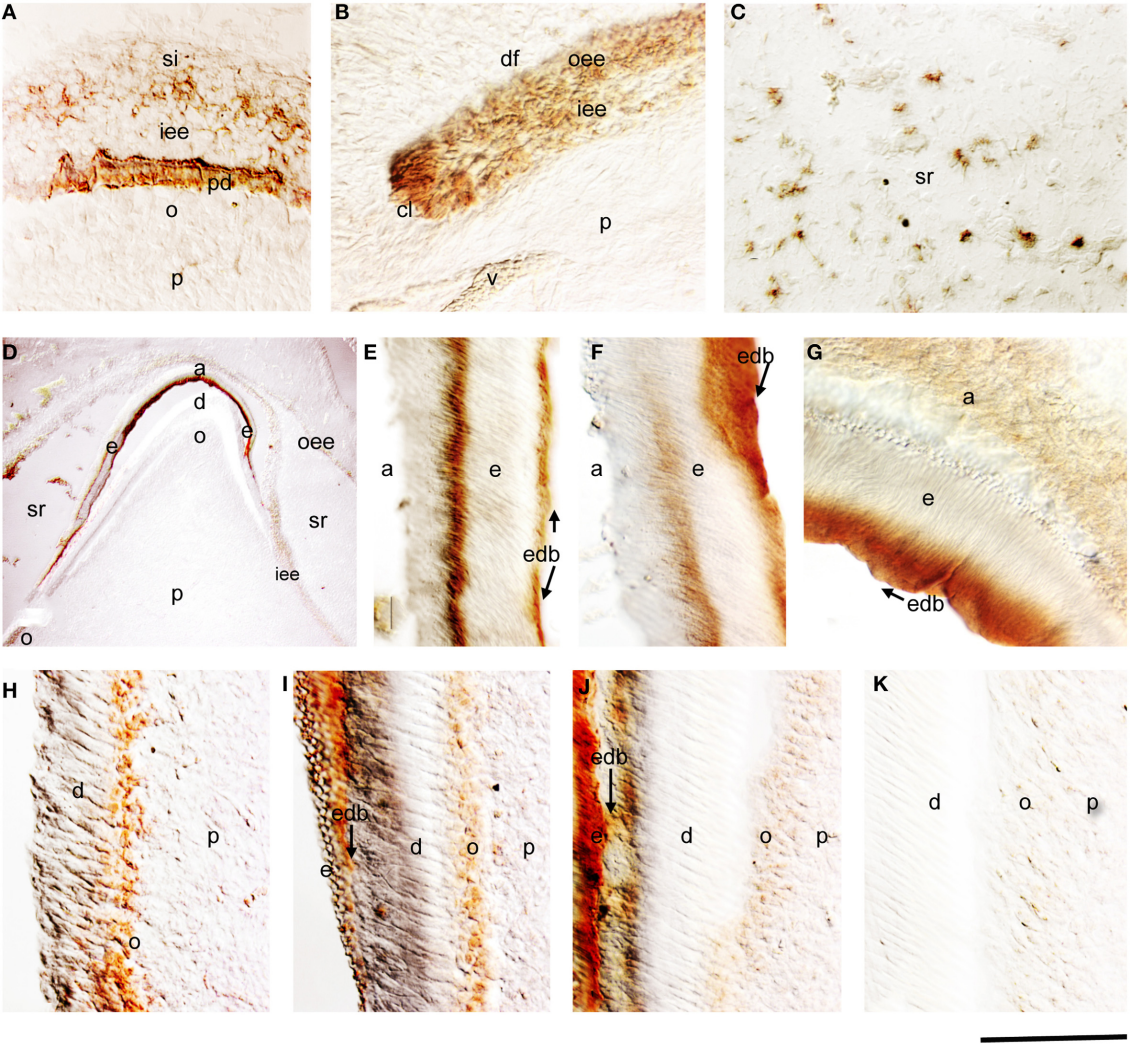
**Amelogenin immunostaining in developing deciduous human teeth. (A)** Cusp area of a deciduous human molar at the early bell stage of development (18 g.w.). Amelogenin is detected in cells of the inner enamel epithelium (iee) and stratum indermedium (si) overlying the predentin (pd), which is also positive for amelogenin protein. **(B)** Cervical loop area of a deciduous human molar (18 g.w.). Amelogenin immunoreactivity is evident in cells of the outer enamel epithelium (oee) and iee. **(C)** Amelogenin staining in some cells of the stellate reticulum (sr) of a 18 g.w. deciduous human molar. **(D)** Cusp area of a deciduous human first incisor at the late bell stage of development (30 g.w.). Strong amelogenin staining is found in enamel (e). A weaker amelogenin reactivity is detected in ameloblasts (a), iee, oee, and sr. In the dental pulp (p) the amelogenin staining is only found in newly differentiated odontoblasts (o). **(E–G)** High magnifications showing positive and negative zones of amelogenin staining in enamel (zebra-like pattern). Note the strong labeling at the dentin-enamel border (edb; arrows). **(H–K)** High magnifications showing the gradient of amelogenin immunoreactivity in odontoblasts according to their maturation degree. Newly differentiated odontoblasts exhibit strong staining **(H)**, which decreases once enamel deposition starts **(I,J)** and completely disappears from more mature odontoblasts **(K)**. Additional abbreviations: df, dental follicle; v, vessels. Scale bar: 50 μm **(A–C, E–K)**, and 80 μm **(D)**.

From the 18th to 21st gestational week (g.w.), the dental epithelium acquires a bell-shaped structure. Dentinogenesis has already started at the tip of the cusps (Figure 1A). Pulp cells adjacent to the inner enamel epithelium (IEE) layer differentiate into odontoblasts, which start to secrete the organic matrix components of predentin. Amelogenin staining was detected in differentiating IEE cells that have acquired a preameloblastic phenotype at the tip of the cusp (Figure 1A). While in the dental pulp the amelogenin immunoreactivity was absent in dental pulp cells, a strong staining was detected in the newly deposited predentin (Figure 1A). Amelogenin staining was also found in proliferating IEE cells of the cervical areas (Figure 1B), and in few stratum intermedium (SI) and stellate reticulum (SR) cells (Figure 1C).

During the 30th g.w., the tooth germs reach the late bell stage of their development. Preameloblasts contacting the predentin differentiate into ameloblasts, which synthesize the enamel matrix proteins. Strong amelogenin staining was detected in the deposited enamel (Figure 1D). SR cells still express low amounts of amelogenin sporadically (Figure 1D). Cells of the outer enamel epithelium (OEE) were also immunostained for amelogenin but in a lesser extend (Figure 1D). The staining in the enamel was not homogenous and showed a zebra-like pattern: the initial intense staining decreased and then increased again, thus creating positive and negative to amelogenin zones (Figures 1D–F). Amelogenin immunoreactivity was detected in functional ameloblasts (Figure 1G). In the pulp, a strong amelogenin immunoreactivity was detected in the differentiating odontoblasts (Figures 1D,H–J). The staining became very weak in functional odontoblasts and progressively disappeared, while it was absent from dental pulp fibroblasts (Figures 1H–K).

### Amelogenin expression in injured and carious permanent human teeth

**Figure 2:**
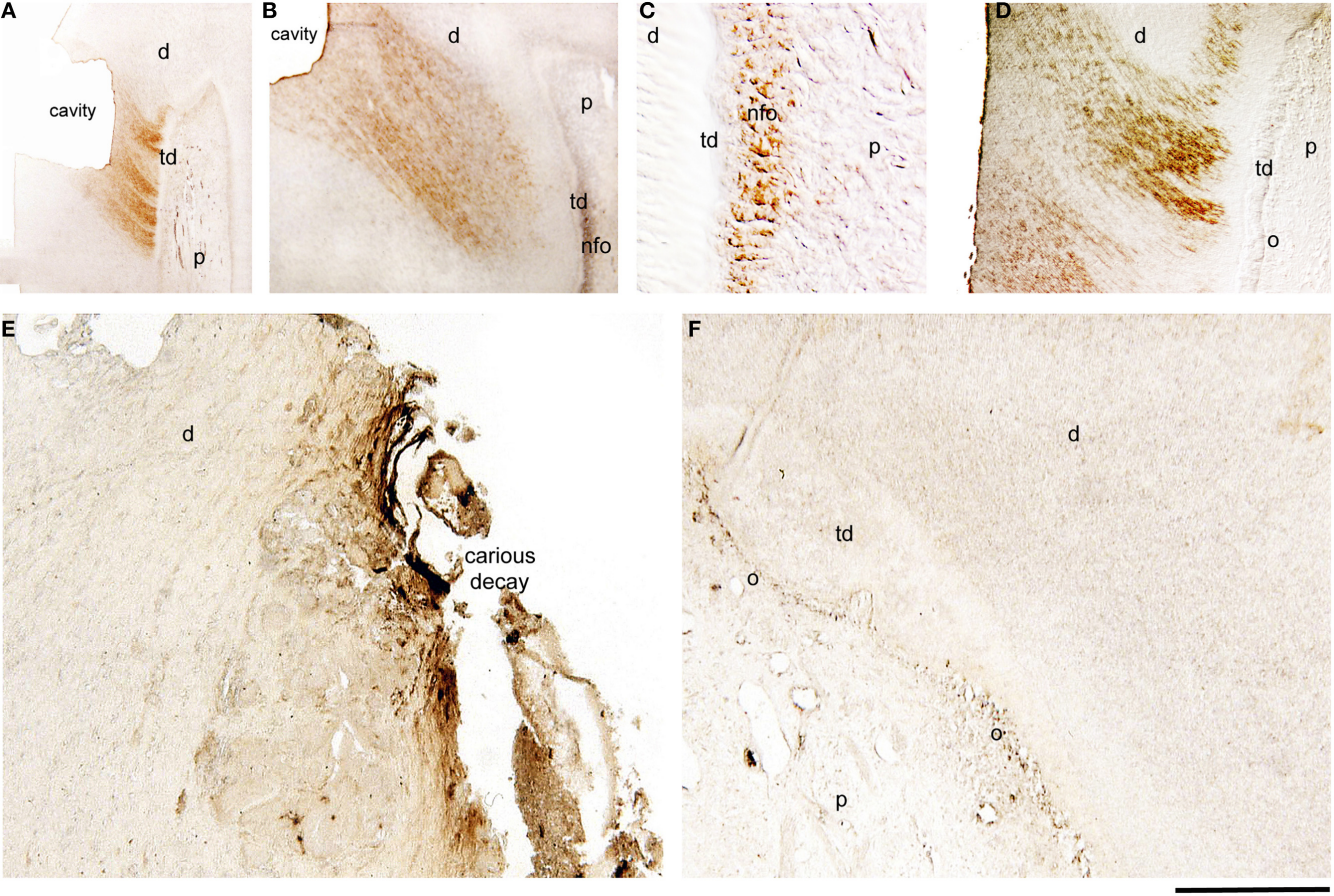
**Immunohistochemical localization of amelogenin in sections of injured and carious permanent human teeth**. Cavitated **(A–D)** and carious **(E,F)** teeth. **(A)** Amelogenin staining in dentinal tubuli facing the class V cavity preparation 4 weeks post-operation. **(B)** Strong amelogenin labeling in newly-formed odontoblasts (nfo) that produce the tertiary dentin (td) 7 weeks post-injury. The staining is also detected in dentinal tubuli facing the injury site. **(C)** Higher magnification of the previous figure, showing amelogenin immunoreactivity in newly formed odontoblasts. **(D)** Amelogenin labeling in dentinal tubuli facing the injury 9 weeks post-operation. Note that the staining is absent from more mature newly-formed odontoblasts (o). **(E)** In carious teeth, amelogenin immunoreactivity is detected in the dentin at the surface of the dental decay. **(F)** Amelogenin in odontoblasts facing the carious lesions that produce the tertiary dentin. Additional abbreviations: d, dentin; p, pulp. Scale bar: 200 μm **(A)**, 100 μm **(B)**, 50 μm **(C)**, and 80 μm **(D–F)**.

Amelogenin immunoreactivity was absent from odontoblasts and pulp fibroblasts of intact permanent human teeth (data not shown). After deep cavity preparations, new odontoblasts, which substitute the disintegrated by the injury odontoblasts, start to produce the reparative or tertiary dentin between the 4th and 9th week postsurgery. In cavitated permanent teeth, amelogenin staining was detected in dentinal tubuli that are related to the injury site (Figures 2A,B,D) and in newly-formed odontoblasts that produce the reparative dentin (Figures 2B,C). Amelogenin staining was detected only in these odontoblasts 4 weeks post cavity preparation (Figures 2B,C). In contrast, amelogenin immunoreactivity become negative in newly-formed odontoblasts 9 weeks after the cavity preparation (Figure 2D). Amelogenin immunostaining was absent in odontoblasts located far away from the injury site (Figure 2B).

In carious teeth, odontoblasts facing the carious front increase their activity and start to produce reactionary dentin (hypercalcification) to protect dental pulp integrity. In carious permenant human teeth, amelogenin staining was observed in the infected demineralized dentin at the border with the carious enamel (Figure 2E), as well as in odontoblasts producing the tertiary dentin (Figure 2F). Dental pulp fibroblasts and odontoblasts in distance of the pathological sites were negative for amelogenin (Figure 2F).

### Amelogenin expression in cultured human dental pulp cells *in vitro*

**Figure 3:**
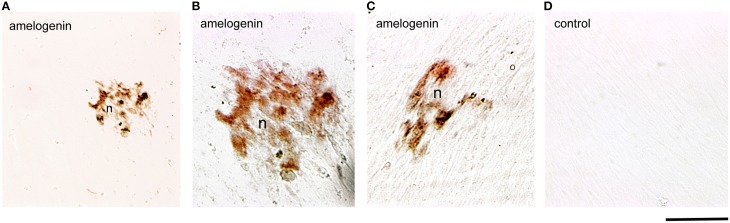
**Amelogenin immunoreactivity in human dental pulp cells cultured *in vitro***. **(A–C)** Amelogenin staining is observed in pulp cells implicated in the formation of the nodules (n) after β-glycerophosphate treatment. All other confluent pulp cells do not exhibit amelogenin labeling. **(D)** Confluent pulp cells cultured without β-glycerophosphate are negative for amelogenin. Scale bar: 50 μm **(A)**, and 35 μm **(B–D)**.

Dental pulp cells were isolated from healthy developing human teeth that were extracted for orthodontic reasons. Cells were cultured for 4 weeks either in the presence or absence of β-glycerophosphate to promote odontoblast differentiation and matrix formation. At the 4th week of culture, deposition of mineral crystals was detected only in the cultures of dental pulp cells treated with β-glycerophosphate (Figures 3A–C). Amelogenin immunoreactivity was detected in the restricted number of dental pulp cells that started to form mineral nodules (Figures 3A–C). Dental pulp cells that do not form nodules (Figures 3A–C), as well as cells cultured in the absence of β-glycerophosphate, were negative for amelogenin (Figure 3D).

## Discussion

Amelogenin is the main enamel matrix protein, comprising more than 90% of the extracellular matrix in the secretory stage of amelogenesis (Fincham and Simmer, 1997). Although strong evidence exists for the role of amelogenin in enamel formation and pathology, much less is known regarding its expression and function during the formation and regeneration of other dental and non-dental tissues. Accumulated evidence suggests that amelogenin could act as a signaling molecule in these tissues. It has been also proposed that amelogenin may influence the fate of mesenchymal progenitor cells (Veis et al., 2000). Concerning the tooth, previous studies using animal models have shown that amelogenin is also localized in root epithelium (Fong and Hammarström, 2000; Fukae et al., 2001; Janones et al., 2005), suggesting additional roles for amelogenin in root formation. Indeed, a mixture of porcine enamel proteins, also including amelogenin, is able to stimulate cementogenesis *in vivo*. Amelogenin has been also detected in dentin by immunohistochemistry (Inai et al., 1991; Bronckers et al., 1993; Nanci et al., 1998). To explain these results, it has been proposed that amelogenin originating from the ameloblastic layer can diffuse and translocate into both the odontoblast layer and dentin (Nakamura et al., 1994) that is clearly observed in conditions in which the amounts of secreted amelogenin is significantly increased (Massa et al., 2006). However, more recent studies using *in situ* hybridization techniques (Papagerakis et al., 2003) have shown that amelogenin is also endogenously expressed by odontoblasts.

Limited information exists about amelogenin expression in human dental tissues. Only one study has provided partial information on amelogenin expression in the dentin of human tooth germs, well before the initiation of enamel matrix formation (Ye et al., 2006). The present findings show that the amelogenin protein is expressed in both epithelial and mesenchymal components of the developing human tooth germs. At the early bell stage, amelogenin is detected only in dental epithelial cells and in predentin. At the late bell stage, newly differentiated odontoblasts started to express amelogenin, but this expression is progressively downregulated following the maturation gradient of odontoblasts. Although amelogenin is not observed in mineralized dentin, an interesting zebra-like pattern of amelogenin protein distribution is observed in the enamel: two amelogenin-positive layers, one near the dentin-enamel border and another at the mineralization front, are splited by an amelogenin-negative layer. This wave-like pattern of amelogenin distribution in enamel could be due to differential amelogenin processing during the secretory stage. It could also be correlated to specific clock genes that operate during odontogenesis. Indeed, we showed that the total amount of enamel secreted proteins follows a biological rhythm (Simmer et al., 2010), and that clock genes regulate the expression of enamel proteinases, kallikrein 4 and MMP20 (Athanassiou-Papaefthymiou et al., 2011). Consistently, previous findings demonstrated that clock genes are expressed by ameloblasts and odontoblasts during tooth development and that amelogenesis is under circadian control (Zheng et al., 2011, 2013). Taken together, these data suggest that clock genes play key roles in amelogenin synthesis, secretion and processing.

Amelogenin expression is also detected in cells of the OEE and SI. Cells of the outer dental epithelium contribute to the generation of Hertwig's sheath epithelium during dental root formation. The present findings in human teeth are consistent with previous data in rodents showing expression of amelogenin in the epithelium of the root (Bronckers et al., 1993; Fong and Hammarström, 2000) and in SI, which is stained positive for X-gal in bovine Amel promoter-lacZ transgenic mice (Adeleke-Stainback et al., 1995). It is thus possible that amelogenin has an additional role in the differentiation of root epithelial cells that give rise to cementoblasts.

In dental mesenchyme, amelogenin expression is only found temporally in healthy young odontoblasts where it is secreted in predentin. Amelogenin expression is terminated in differentiated odontoblasts secreting dentin. Although, it is unknown the role of amelogenin in this short developmental window we suggest that it may play a signaling role which contributes to odontoblast differentiation and initiation of dentin formation. In contrast, amelogenin is not detected in mature odontoblasts or pulp fibroblasts during normal development.

**Figure 4:**
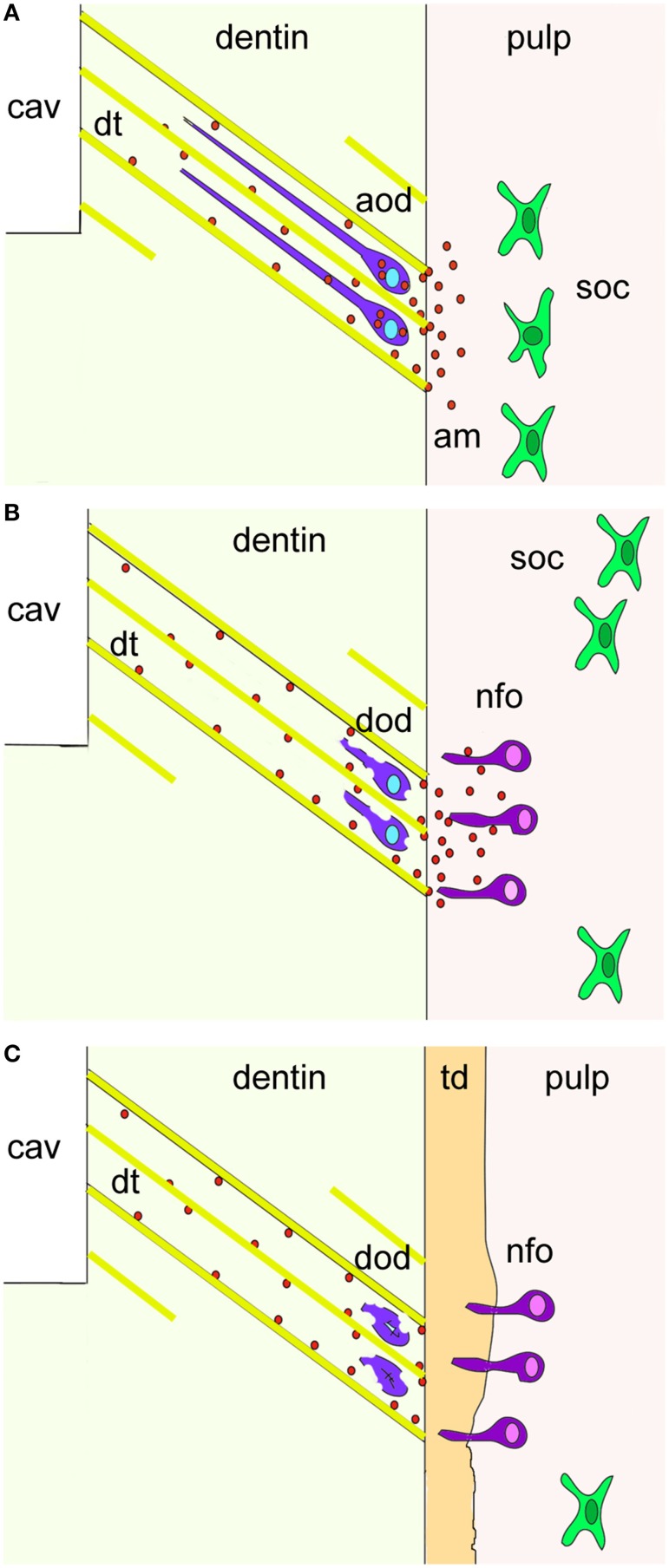
**A hypothetical model summarizing the cellular events after dental injury**. Cavity preparation (cav) leads to the apoptosis of odontoblasts due to their aspiration into the dentinal tubuli (dt; yellow color). Amelogenin molecules (am; red spots) secreted by aspirated odontoblasts (violet color) before undergo apoptosis activate pulp progenitor cells such as sub-odontoblastic cells (soc; green color) to differentiate into new-odontoblasts (nfo; dark pink color) that start to form the tertiary dentin (td; orange color). Additional abbreviations: aod, apoptotic odontoblasts; dod, disintegrated odontoblasts.

Mature odontoblasts can be damaged by deep cavity preparations involving the dentin or by caries affecting enamel and dentin. The damaged odontoblasts are replaced by odontoblast progenitors, which produce a reparative dentin matrix close to the injury site (About et al., 2000b; Heymann et al., 2002). Although it is known that the quantity and the quality of the deposited reparative dentin matrix varies among different individuals and types of injury, the molecular players of these patho-physiological events remain largely unknown (Mitsiadis and Rahiotis, 2004). The present findings show that newly formed odontoblasts facing the injury and carious sites express *de novo* amelogenin, suggesting that amelogenin is involved in the dentin repair process. Interestingly, amelogenin protein is preferentially detected in the dentin directly under the wounded site and in the carious front. It has been shown that during cavity preparation the damaged odontoblasts are soaked into the dentinal tubuli and thereafter die by apoptosis. Thus, it is possible that these damaged odontoblasts re-express amelogenin before undergoing apoptosis (Figure 4). It is also conceivable that the newly formed odontoblasts may secrete amelogenin into the dentin matrix as a signal of initiating reparative dentin formation.

The significance of amelogenin in enamel formation has been confirmed with the generation of null mutant mice (Gibson et al., 2001). Furthermore, in a rat model of rickets, the enamel structure and phenotype closely resembles to the hypoplastic form of enamel that is observed in amelogenin mutations (Papagerakis et al., 1999). As expected, *amelogenin* mRNA was down-regulated in ameloblasts of rachitic rat teeth, but surprisingly, the gene was upregulated in odontoblasts that produce a hypomineralized form of dentin, thus suggesting an inverse regulation by vitamin D in odontoblasts. Consistently, significant differences between enamel and dentin phenotypes have been found in patients with hereditary vitamin D-resistant rickets (HVDRR). In these patients the concentration of phosphorus in the dentin was extremely low while the concentration of both calcium and phosphorus in the enamel were equal to those of normal teeth (Hillmann and Geurtsen, 1996). Furthermore, formation of extensive reactionary dentin has been observed at the pulp horn of teeth in patients with HVDRR suggesting odontoblasts hyperactivity. Thus, amelogenin expression levels in ameloblasts vs. odontoblasts can be significant different under pathological conditions and these variances may correlate with cell-specific functions. In fact, the amelogenin that is detected in odontoblasts during reactionary dentin formation may represent a response to hypomeniralization triggered by dental caries similar to the effects described in vitamin D-caused hypomineralization. Thus, the up-regulation of amelogenin in odontoblasts observed in rickets or caries suggests a potential role for amelogenin in reactionary dentin matrix formation and mineralization.

Growth factors such as bone morphogenetic proteins (BMPs) and fibroblast growth factors (FGFs), and proteinases, including matrix metalloproteases (MMPs) and cysteine cathepsins (CCs), are found in dentin. During tooth repair, these molecules are released from the damaged dentin and diffuse to reach adjacent cells, thus promoting their differentiation into odontoblasts that will form the reparative dentin. The correct balance between these molecules is critical for preserving dental pulp integrity. Fast carious progression has been correlated with high levels of MMPs and CCs expression in dentin (Vidal et al., 2014). BMP and FGF signals are known to induce amelogenin expression (Catón et al., 2009; Cao et al., 2013) while MMPs can cleave amelogenin (Khan et al., 2013). Cleavage of amelogenin by MMPs accelerates mineralization, a process that also depends on mineral ions (Khan et al., 2013). Thus, MMPs and growth factors released by the injured dentin may be instrumental in inducing *de novo* amelogenin expression, secretion, and cleavage.

Understanding the molecular interplay among growth factors, proteases, and amelogenin during dentin repair will certainly advance the field of dental materials guiding the development of therapies to effectively control dental decay. In fact, several efforts are being made to include amelogenin within dental materials with the aim to achieve a better dentin repair (Goldberg et al., 2009). However, clinically applicable protocols are still missing. Nevertheless, the present study suggests that amelogenin plays important roles not only in ameloblasts and odontoblast differentiation, but also in matrix deposition, mineralization, and maturation during development and repair of human teeth.

### Conflict of interest statement

The authors declare that the research was conducted in the absence of any commercial or financial relationships that could be construed as a potential conflict of interest.
